# Supplementary material for: KAP1 is an antiparallel dimer with a functional asymmetry
Source: Life Sci Alliance. 2019 Aug 19;2(4):e201900349. doi: 10.26508/lsa.201900349 (PMC6701479; doi:10.26508/lsa.201900349)
Supplement: Supplementary file 1 [file LSA-2019-00349_TableS1.doc]

**Supplementary Table 1: SAXS parameters**

|  | KAP1 FL | ΔKAP1 | RBCC |
| --- | --- | --- | --- |
| **SASPDB accession code** | SASDEV6 | SASDER7 | SASDEW6 |
| **Data collection** | | | |
| Instrument | ESRF BM29 | | |
| Beam size at sample (µm) | 700x700 | | |
| Wavelength (Å) | 0.992 | | |
| q range (Å^-1^) | 0.0025-0.500 | | |
| Detector | Pilatus 1M | | |
| Detector distance (m) | 2.867 | | |
| Exposure (s per image) | 1 | | |
| Column | Superose 6 Increase 10/300 | | |
| Flow rate (ml/min) | 0.5 | | |
| Sample volume (µl) | 100 | | |
| Sample concentration (mg/ml) | 15 | 12 | 12 |
| Temperature (K) | 293 | | |
|  | | | |
| **Structural parameters** | | | |
| R_g_ (Å) Guinier | 90 | 89 | 83 |
| R_g_ (Å) P(r) | 95 | 95 | 89 |
| R_gc_ (Å) | 35.8 | 38.8 | 20.2 |
| D_max_ (Å) | 380 | 380 | 370 |
| Porod volume (Å^3^) | 710000 | 853000 | 381000 |
|  | | | |
| **Molecular mass determination** | | | |
| Theoretical MW (kDa) | 183 | 175 | 92 |
| MALLS MW (kDa) | 190 | 183 | 88 |
| DATPOROD MW (kDa) | 444 | 533 | 238 |
| DATVC MW (kDa) | 255 | 290 | 117 |
| DATMOW MW (kDa) | 247 | 321 | 145 |
|  |  | | |
| **Data analysis software** |  | | |
| Data reduction | PRIMUS & ScÅtter | | |
| *Ab initio* modelling | GASBOR | | |
| Homology modelling | Swiss Model/I-tasser | | |
| Computation of model fitting to data | Pepsi-SAXS | | |
| 3D graphics representation | Pymol | | |

**References**

1. Schuck P (2000) Size-distribution analysis of macromolecules by sedimentation velocity ultracentrifugation and lamm equation modeling. *Biophys J* 78: 1606–1619.

2. Konarev PV, Volkov VV, Sokolova AV, Koch MHJ, Svergun DI (2003) PRIMUS: a Windows PC-based system for small-angle scattering data analysis. *J Appl Cryst,* 36: 1277–1282.

3. Rambo RP (2015) Resolving Individual Components in Protein-RNA Complexes Using Small-Angle X-ray Scattering Experiments. *Meth Enzymol* 558: 363–390.

4. Bernadó P, Mylonas E, Petoukhov MV, Blackledge M, Svergun DI (2007) Structural Characterization of Flexible Proteins Using Small-Angle X-ray Scattering. *Journal of the American Chemical Society* 129: 5656–5664.
